# The role of lyophilized Aloe vera gel in growth, liver health, redox homeostasis, and enhancing resilience of broiler chicks to high-temperature conditions

**DOI:** 10.1007/s11250-026-04862-9

**Published:** 2026-02-14

**Authors:** Walid S. Habashy, Ayman M. Morsi, Osama A. Elghalid, Ahmed M. Abd El-Hady, Asmaa Sh. El-Naggar

**Affiliations:** 1 Department of Animal and Poultry Production, Faculty of Agriculture, Damanhour University, Damanhour, 22516 Egypt; 2 Poultry Production Department, Faculty of Agriculture (El-Shatby), Alexandria University, Alexandria, 21546 Egypt

**Keywords:** Aloe vera, Antioxidant, Broiler, Growth promoters, Heat stress, Liver

## Abstract

The Egyptian summer presents a challenging environment for poultry production due to elevated temperatures and humidity, which adversely affect bird performance and health. This study aimed to assess the in vivo antioxidant potential of varying dietary levels of lyophilized Aloe vera gel (LAV) and to examine its influence on growth performance, liver health, redox homeostasis, and enhancing resilience of broiler chickens reared under high-temperature conditions. A total of 180 unsexed Ross 308 broiler chicks, seven days old, were randomly assigned to six different diets (0, 25, 50, 100, 150, and 200 mg LAV/kg of feed), with each diet group having 30 birds divided into five replicates of six chicks each. The trial spanned five weeks. Results demonstrated that dietary LAV supplementation significantly enhanced body weight gain and improved the digestibility of crude protein, lipids, and dry matter. Hematological parameters, including red blood cell count, hemoglobin concentration, and packed cell volume, were also positively affected. LAV further improved the lipid profile and markedly elevated serum antioxidant indices, superoxide dismutase, glutathione, glutathione peroxidase, and total antioxidant capacity, while significantly reducing malondialdehyde concentrations, indicating lower oxidative stress levels. In conclusion, dietary inclusion of lyophilized Aloe vera gel improved productive performance, hepatic and renal function, and antioxidant status in a dose-responsive manner. These enhancements contribute to improved oxidative balance and may support better health and performance in broilers exposed to high environmental stress.

## Introduction

Poultry meat is widely recognized as a valuable source of high-quality nutrients due to its favorable nutritional profile, affordability, and broad accessibility, making it a preferred protein source among consumers (Hassan et al. 2020). Broiler chickens account for over 90% of the global poultry population (Augère-Granier 2019), with feed costs comprising approximately 65–70% of total production expenses. To reduce these high feed-related costs, nutritionists are increasingly exploring the use of agricultural by-products, unconventional feed ingredients, and natural growth-promoting additives as sustainable and cost-effective alternatives (Fadzlin et al. 2020; Abd Elghafar et al. 2024). The broiler industry represents a major pillar of the Egyptian agricultural sector, supported by strong domestic demand, rapid population growth, and increasing per capita consumption of poultry meat (Fotouh et al. 2024). Broiler production plays a vital economic role by contributing to national income and creating substantial employment opportunities (Hamiyanti et al. 2023).

Antibiotics were historically incorporated into poultry diets to support growth and prevent disease. However, their extensive and prolonged use has led to antimicrobial resistance, accumulation of drug residues in tissues, and disruption of gut microbiota, raising food safety concerns (El-Ghousein and Al-Beitawi 2009; Abd El-Hady et al. 2022). Consequently, regulatory restrictions, including the EU ban on antibiotics as growth promoters in 2006, have driven the search for non-antibiotic growth promoters (Yadav et al. 2017; Lees et al. 2021). Recent studies emphasize the potential of phytogenic compounds, including medicinal herbs, plant extracts, and essential oils, to serve as natural alternatives to antibiotics by improving gut health, enhancing immune responses, and mitigating disease risk (Rashid et al. 2024; Hailat et al. 2024; Hayajneh et al. 2024). These phytochemicals are particularly promising for addressing drug resistance issues while maintaining poultry productivity.

Heat stress poses a serious challenge to broiler welfare and the economic sustainability of the poultry industry. Under climate change conditions and rising ambient temperatures, effective mitigation of heat stress has become essential, particularly in hot regions such as Egypt during summer months (Farida et al. 2022). Heat stress negatively affects growth performance, immunity, and meat quality, increases susceptibility to viral and bacterial infections, and ultimately reduces productivity and profitability in broiler production systems (Elmeligy et al. 2024). Summer heat stress in Egypt causes marked economic losses in broiler (fattening) production by reducing feed intake and growth rate, worsening feed conversion, increasing mortality and carcass downgrades, and raising veterinary and cooling costs effects which together depress farm profitability, especially in open-shed systems common in Egypt (Abd-El Hamed et al., 2025).

Oxidative stress in poultry occurs when reactive oxygen species exceed antioxidant defenses (e.g., glutathione peroxidase, catalase, superoxide dismutase), causing lipid peroxidation, protein and DNA damage, and cellular disruption. In the gut, it impairs barrier integrity, microbiota balance, and nutrient absorption, reducing feed efficiency, growth, and disease resistance. Systemically, it weakens immunity, lowers antibody production, and disrupts metabolic and organ functions (Li et al. 2024).

The overuse of antibiotic growth promoters in poultry has raised major concerns, including the emergence of antibiotic-resistant bacteria, accumulation of drug residues in tissues, and disruption of intestinal microbiota (El-Ghousein and Al-Beitawi 2009). Consequently, non-antibiotic growth promoters have emerged as promising alternatives, promoting beneficial gut microbiota, enhancing digestive stability, and improving feed efficiency (Yadav et al. 2017). Natural antioxidants in medicinal plants mitigate oxidative stress, enhance immunity, and improve growth performance (Botsoglou et al., 2001). Garlic enhances antioxidant enzymes (glutathione peroxidase, superoxide dismutase), reduces lipid peroxidation, supports gut integrity, modulates immunity, and improves growth and blood lipid profile (Abd El-Ghany, 2024). Green tea provides polyphenols, flavonoids, and vitamins that scavenge free radicals, reduce oxidative stress, and enhance performance and health parameters in broilers and layers (Abd El-Ghany, 2025).

Overall, Phyto-biotics strengthen endogenous antioxidant defenses, minimize tissue oxidative damage, maintain gut barrier integrity and microbiota homeostasis, and improve disease resistance, nutrient absorption, and productive performance (Abd El-Hady et al. 2019). This highlights the pivotal role of functional feed additives, including yeast derivatives, organic acids, and propolis, in promoting sustainable poultry nutrition (Abd El-Ghany et al., 2024; 2025). Also, among these phytogenic compounds, such as *Aloe vera*, are gaining increasing attention for their natural origin and broad-spectrum functional properties.

*Aloe vera* (AV) is a semi-tropical plant from the Aloe genus, widely known for its medicinal value (Nandal and Bhardwaj 2012). The name originates from the Arabic “Alloeh” (shining bitter substance) and Latin “vera” (true) (Surjushe et al. 2008). It has been traditionally used across various civilizations, including ancient Egypt and Greece (Marshall 1990). The commercially important species are *Aloe barbadensis* and *Aloe arborescens*, with the leaves being the main source of their bioactive compounds (Vogler and Ernst 1999). AV contains over 200 active phytochemicals, including antioxidants like polyphenols, indoles, and alkaloids (de Oliveira et al. 2018). These compounds provide a range of biological activities, such as antibacterial, antiviral, antifungal, anti-inflammatory, antidiabetic, and immune-boosting effects. In poultry science, AV has gained interest as a natural growth promoter and antioxidant (Jalal et al. 2019; Almasry et al. 2023). Its inclusion in broiler diets has been linked to improved immune function, growth performance, and overall health (Ebrahim et al. 2020).

Given the increasing concerns over oxidative stress and the limitations of synthetic additives, there is an urgent need to explore natural antioxidant alternatives that can boost poultry productivity. Therefore, this study was designed to assess the in vivo antioxidant potential of different inclusion levels of lyophilized *Aloe vera* gel (LAV) and to investigate its effects on growth performance, liver health, redox homeostasis and enhancing resilience in broiler chickens raised under the challenging conditions of the Egyptian summer.

## Materials and methods

The present experiment was conducted at the Animal and Poultry Research Centre (El-Bostan Farm), Faculty of Agriculture, Damanhour University, during the period from August to September 2023. The primary objective was to evaluate the effects of varying dietary inclusion levels of lyophilized *Aloe vera* (LAV) on growth performance, nutrient digestibility, hematological and biochemical blood parameters, and antioxidant status in broiler chickens under high temperature conditions typical of the Egyptian summer. All experimental, including animal handling, housing, and sampling protocols, were reviewed and approved by the Institutional Animal Care and Use Committee (IACUC) of Damanhour University, Egypt (DUFA-2025-3) in accordance with national and international guidelines for the care and use of animals in research.

### Collection and preparation of lyophilized *Aloe vera* gel

Fresh, mature *Aloe vera* leaves were collected from a botanical garden in Abo Homos, Beheira, Egypt. The leaves were about 25–30 cm long and 4–6 cm wide. After washing, the outer green rind was carefully removed using a sterile blade, and the inner gel was separated and homogenized following the method of Almasry et al. (2023). The gel was then freeze-dried (lyophilized) using an SP Scientific VirTis 4KBTZL-105 Benchtop Freeze Dryer at -109 °C and 20 mTorr (millitorr) pressure. The freeze-drying process included freezing, sublimation (primary drying), and desorption (secondary drying), which helped retain the gel’s nutrients and active compounds. The lyophilized *Aloe vera* (LAV) powder was stored at room temperature for later use (Lee and Lee 2009; Anadani and Vyas 2017). Table 1 shows the main bioactive components found in the lyophilized gel.

**Table 1:** Proximate chemical composition, total flavonoids, and saponins content of lyophilized *Aloe vera* gel

Proximate analysis of lyophilized Aloe vera (LAV)	%
Moisture	2.10
Crude protein	15.20
NFE	60.10
Total sugars	14.30
Ash	3.10
Crude fiber	2.90
Crude oils	2.30
**Total flavonoids and saponins**	
Flavonoids	3.31
Saponins	4.11

NFE: nitrogen-free extract

### Experimental birds

A total of 180 unsexed *Ross 308* broiler chicks, aged 7 days, were utilized in this study. At the onset of the experiment, chicks were individually weighed to the nearest gram, wing-banded, and randomly allocated to six dietary treatments, each comprising 30 birds. Each treatment was further divided into five replicates, with six chicks per replicate. The initial body weights of the chicks were homogeneous, ranging from 144.0 to 146.0 g. The composition of the formulated diets is presented in Table 2. Diets were formulated to meet the nutrient requirements of the National Research Council (1994) with respect to protein and energy levels, vitamins, and minerals, as required at different phases of growth. The first treatment was fed the basal diet without any supplementation (control), while the 2nd, 3rd, 4th, 5th, and 6th treatments received the basal diet supplemented with 25, 50, 100, 150, and 200 mg/kg of LAV, respectively. Feed and clean drinking water were provided *ad libitum* throughout the experimental period (7–35 days).

**Table 2:** Composition and calculated analysis of the basal diet

Ingredients (%)	Starter period (7–21 days)	Grower period (22–35 days)
Yellow Corn	54.00	59.00
Soybean Meal (46%)	27.00	21.20
Full-fat soya,	5.00	7.00
Gluten (60%)	8.00	7.00
Soya oil	1.50	1.30
Mono Calcium Phosphate	1.65	1.65
Limestone	1.75	1.75
L-lysine	0.25	0.25
DL–methionine	0.20	0.20
Salt (Na Cl)	0.35	0.35
Premix*	0.30	0.30
Total	100.0	100.0
**Calculated analysis**		
Crude Protein, %	22.9	21.4
Metabolic energy, kcal/kg	3042	3103
Crude Fiber, %	2.70	2.70
Ether extract, %	4.10	4.45
Calcium, %	1.01	1.01
Phosphorus available%	0.50	0.51
Methionine%	0.66	0.61
Lysine %	1.33	1.25
Methionine + Cystine %	1.05	0.98

*: Each kg of vitamin and mineral mixture contains 12 M IU vitamin A; 5 M IU D3; 80,000 mg E; 4000 K mg; 4000 mg B1; 9000 mg B2; 4000 mg B6; 20 mg B12; 15,000 mg pantothenic acid; 60,000 mg Nicotinic acid; 2000 mg Folic acid; 150 mg Biotin; 400,000 mg Choline Chloride; 15,000 mg Copper sulphate; 1000 mg calcium Iodide; 40,000 mg ferrous sulphate; 100,000 mg Manganese oxide; 100,000 mg Zinc oxide and 300 mg Selenium selenite

### Housing and management

During the preliminary period, all chicks obtained from a commercial hatchery were reared under thermoneutral conditions in an environmentally controlled facility. Thereafter, they were transferred to a semi-open house equipped with two exhaust fans to ensure adequate ventilation. The birds were housed in galvanized wire battery cages with standard dimensions of 65 × 65 × 40 cm (length × width × height), suspended 50 cm above floor level. Uniform management practices, hygiene protocols, and environmental conditions were maintained consistently throughout the experimental period. Broiler chicks were vaccinated against major poultry diseases in a well-structured immunization schedule under Egyptian production conditions. The Hitchner B1 strain at 7 days of age and the La Sota strain at 18 administered via drinking water to control Newcastle Disease. Vaccination against Infectious Bursal Disease (Gumboro) was performed at 12 days via drinking water. The Colon30 vaccine was applied at 28 days. This schedule was elaborated to provide maximum immune protection during the experimental period. A standard lighting program was implemented: 23 h of light per day during the first week, followed by 20 h of light daily until the end of the trial. Upon arrival, the chicks were brooded at 33 °C for the first two days, with the temperature subsequently reduced by 0.5 °C per day until reaching day 6. From day 7 to day 35, all treatments were exposed to natural ambient temperature conditions prevailing in the facility (August to September), with temperature and relative humidity monitored periodically.

### Temperature-humidity index calculation

Ambient temperature and relative humidity were monitored daily from August to September using an automatic data logger (Datalogger-Log 100/10, Germany). The highest and lowest values recorded each day were used to calculate daily averages, as presented in Table 3. Inside the poultry house, a psychrometer was used to measure dry-bulb (Tdb) and wet-bulb (Twb) temperatures, with an accuracy of 0.1 °C. The Temperature-Humidity Index (THI) was calculated using the formula adapted from Tao and Xin (2003):

**Table 3:** Overall means of temperature ͦ°C (Temp), relative humidity % (RH), and temperature-humidity index (THI)

Month		Temp ͦ°C		RH %	THI
		Tdb	Twb		
August	Second week	28.5	21.7	70.0	27.48
	Third week	32.4	22.4	69.0	30.90
	Fourth week	31.2	20.3	71.0	29.56
September	Fifth week	31.9	21.1	73.0	30.28
**Overall means**		**31.0**	**21.3**	**70.75**	**29.54**

$${\text{THI = 0}}{\text{.85 }} \times {\text{ Tdb + 0}}{\text{.15 }} \times {\text{ Twb,}}$$

where Tdb (dry-bulb temperature) and Twb (wet-bulb temperature) are expressed in degrees Celsius. This index was used to evaluate the level of heat stress experienced by broiler chickens under summer conditions.

### Growth performance traits

Body weight (BW) of broiler chicks was measured at two critical periods: at the start of the experiment (day 7) and at the end of the experiment (day 35). No intermediate weekly measurements were carried out, since the main interest was in the overall growth during the starter and grower periods. Feed consumption (FC) and weight gain (BWG) were monitored, and the feed conversion ratio (FCR) was calculated as the ratio of feed consumed (g) to weight gained (g). Overall performance was further assessed using the European Production Efficiency Index (EPEI), calculated using the following formula:

$${\text{EPEI = (BW }} \times {\text{ SR) / (PP }} \times {\text{ FCR) }} \times {\text{ 100,}}$$

Where: BW = Final body weight (kg), SR = Survival rate (%) = 100 – mortality (%), PP = Production period (days), FCR = Feed conversion ratio (kg feed/kg gain). This index provides an integrated measure of growth performance, feed efficiency, and survivability. Mortality was recorded daily, and all dead chicks were documented for each treatment group throughout the experimental period. The mortality rate (MR) was calculated by dividing the number of dead birds by the total number of chicks at the start of the experiment: MR (%) = (Number of dead chicks/Initial number of chicks) × 100.

### Apparent digestibility trial

At the end of the experiment, a digestibility trial conducted using 30 chicks (five birds per treatment). Each bird was housed individually in specialized metabolism cage. After a 4-day adaptation period, total excreta were collected over the next 3 days. Feed intake was recorded accurately, and daily droppings were collected, oven-dried at 60–70 °C for 24 h, ground, and stored for chemical analysis. Apparent nutrient digestibility coefficients for crude protein (CP), ether extract (EE), crude fiber (CF), dry matter (DM), and ash were determined according to standard procedures outlined by the Association of Official Analytical Chemists (A.O.A.C., 2006).

### Blood constituents

At five weeks of age, blood samples (~ 3 mL) were collected from five randomly selected broiler chicks per each treatment via the brachial vein. Samples were drawn into both heparinized and non-heparinized tubes. Clotted blood samples were centrifuged at 4,000 rpm for 15 min to separate the serum, which was stored at − 20 °C for subsequent biochemical analysis. The heparinized blood samples were divided into two portions: one was used immediately for complete blood count (CBC), while the other was centrifuged to separate plasma, which was also stored at − 20 °C for later analysis. All biochemical parameters were determined using standardized commercial colorimetric assay kits, following the manufacturer’s instructions.

### Hemato-biochemical and antioxidant indices

Red blood cell count (RBC), hemoglobin concentration (Hb), and packed cell volume (PCV) were measured using standard hematological procedures as described by Feldman et al. (2000) and Drew et al. (2004). Blood serum was analyzed for total lipids, triglycerides, and total cholesterol using commercial diagnostic kits with a spectrophotometer, following established protocols outlined by Fringes et al. (1972) and Bogin and Keller (1987). High-density lipoprotein (HDL) levels were determined using the method of Warnick et al. (1983), while low-density lipoprotein (LDL) levels were calculated using the formula:

${\text{LDL = Total}}\,{\text{Cholesterol -- }}\left( {{\text{HDL + Triglycerides/5}}} \right),$ as outlined by Davidson et al. (2013).

Liver enzyme activity-including aspartate aminotransferase (AST) and alanine aminotransferase (ALT)-was measured using colorimetric methods according to Reitman and Frankel (1957), while alkaline phosphatase (ALP) activity was determined by the method of Bauer (1982). Kidney function indicators, including creatinine and uric acid, were assessed using the procedures of Husdan and Rapoport (1968) and Patton and Crouch (1977), respectively. Plasma glucose levels were measured using the Trinder (1969) method with commercial kits. Levels of thyroid hormones triiodothyronine (T3) and thyroxine (T4) were evaluated using the radioimmunoassay (RIA) technique described by Thumvijit et al. (2022), and the T3/T4 ratio was calculated to assess metabolic activity.

Serum malondialdehyde (MDA), an indicator of lipid peroxidation, was measured as thiobarbituric acid-reactive substances (TBARS) using the method described by Placer et al. (1966). Total antioxidant capacity (TAC) was determined based on the sample’s ability to neutralize hydrogen peroxide, as described by Koracevic et al. (2001). Blood glutathione (GSH) levels were assessed according to the method of Beutler et al. (1963). The activities of key antioxidant enzymes-glutathione peroxidase (GSH-Px) and superoxide dismutase (SOD)-were evaluated according to Levander et al. (1983) and Nishikimi et al. (1972), respectively.

### Statistical analysis

Data were analyzed using one-way analysis of variance (ANOVA) under the General Linear Model (GLM) procedure in SAS (2006). The statistical model used was:

Y_ij_ = µ + T_i_ + e_ij_, where Y_ij_ is the observed value, µ is the overall mean, T_i_ is the fixed effect of treatment, and e_ij_ is the random error term. Percentage data were transformed using square root or arcsine methods to ensure normality and homogeneity before analysis. Differences between treatment means were compared using Tukey’s test, and statistical significance was declared at *P* ≤ 0.05.

## Results

### Growth performance

Table 4 presents the growth performance data for broiler chicks from day 7 to 35. At the end of the trial, BW was significantly affected by the different levels of LAV in the diet (*P* = 0.0001). All LAV-supplemented groups showed significantly higher BW than the control group, with percentage increases ranging from 16.8% to 18.6%. Although differences between the LAV levels were not statistically significant, all levels of LAV improved overall performance. Similarly, BWG during the entire period (7–35 days) showed a significant positive response to LAV supplementation. The highest BWG was observed in birds receiving 150 mg/kg LAV, with a 20.2% increase compared to the control.

**Table 4:** Impact of different levels of lyophilized *Aloe Vera* supplementation on growth performance of broiler chickens under Egyptian summer conditions

Traits	Control	Aloe vera supplementation (mg)					SEM	*P*-value
		25	50	100	150	200		
IBW (g)	144.5	145.1	144.0	145.8	146.0	145.5	2.03	0.820
FBW(g)	1790^b^	2091^a^	2099^a^	2100^a^	2120^a^	2122^a^	20.21	0.0001
BWG (g)	1645.5^b^	1945.9^a^	1955.5^a^	1954.2^a^	1978.4^a^	1974.5^a^	21.34	0.0001
FC (g)	2904.0	3057.5	3010.2	3052.5	2939.0	2915.0	24.87	0.097
FCR	1.76^a^	1.57^b^	1.54^b^	1.56^b^	1.49^b^	1.48^b^	0.04	0.002
EPEI	272.4^c^	376.3^b^	377.0^b^	380.3^b^	419.8^a^	425.4^a^	8.98	0.001
MR (%)	6.01^a^	4.54^b^	4.21^b^	4.90^b^	4.43^b^	4.98^b^	0.987	0.002

^a, b^ means in the same column followed by different letters are significantly different at *P* ≤ 0.05. SEM: Standard error of mean; IBW: Initial body weight; FBW: Final body weight; BWG: Body weight gain; FC: Feed consumption; FCR: Feed conversion ratio; European Production Efficiency Index, $\:\mathrm{E}\mathrm{P}\mathrm{E}\mathrm{I}=\frac{\mathrm{B}\mathrm{W}\:\left(\mathrm{k}\mathrm{g}\right)\:\mathrm{x}\:\mathrm{S}\mathrm{R}}{\:\mathrm{P}\mathrm{P}\:\mathrm{x}\:\mathrm{F}\mathrm{C}\mathrm{R}}\mathrm{x}\:100$; MR, Mortality rate

From days 7 to 35, there were no significant differences in FC among the treatment groups (*P* = 0.097), indicating that the observed improvements in growth were not due to increased feed intake. However, the FCR was significantly improved by LAV supplementation. Notably, birds fed 150 mg and 200 mg/kg LAV achieved enhanced FCR values, with improvements of 15.3% and 15.9%, respectively, compared to the control. Mortality rates were significantly reduced in all LAV-treated groups compared to the control group. The reductions ranged from 17.1% in the 200 mg group to 29.9% in the 50 mg group, suggesting improved survivability. Additionally, the EPEI improved across all LAV-treated groups, with improvements ranging from 38.1% (25 mg LAV) to 56.2% (200 mg LAV), reflecting enhanced overall productivity and health status.

### Apparent digestibility

As shown in Table 5, the inclusion of LAV to broiler diets significantly improved the digestibility of several nutrients. Crude protein digestibility was significantly higher in all LAV-supplemented groups compared to the control, with the greatest improvement observed in the group receiving 200 mg/kg LAV (67.1%). Similarly, EE digestibility also showed marked improvement, particularly in birds fed 150 mg/kg (72.4%) and 200 mg/kg (73.0%) LAV, compared to the control group. Although crude fiber and apparent ash digestibility showed slight numerical improvements in the LAV-treated groups, these differences were not statistically significant. Dry matter digestibility increased significantly, with the 50 mg/kg group achieving the highest value (67.1%) compared to 60.9% in the control group.

**Table 5:** Impact of different levels of LAV supplementation on the apparent digestibility of nutrients of broiler chickens under Egyptian summer conditions

Traits	Control	Aloe vera supplementation (mg)					SEM	*P* value
		25	50	100	150	200		
Crude protein (%)	63.2^b^	66.9^a^	65.4^a^	66.2^a^	64.8^a^	67.1^a^	1.82	0.001
Ether extract (%)	66.9^b^	72.0^a^	71.7^a^	71.8^a^	72.4^a^	73.0^a^	2.78	0.001
Crude fiber (%)	14.3	15.0	14.9	15.0	14.7	15.1	2.99	0.087
Dry matter (%)	60.9^b^	66.0^a^	67.1^a^	67.5^a^	65.9^a^	64.8^a^	1.56	0.003
Apparent ash (%)	30.2	29.8	31.9	32.4	31.9	29.5	1.41	0.087

^a, b^, means in the same row followed by different letters are significantly different at (*P* ≤ 0.05); SEM, Standard error of mean; LAV, lyophilized *Aloe vera*

### Hematological parameters

The results presented in Table 6 indicate that all groups supplemented with LAV showed significantly higher RBC counts compared to the control group (2.99 × 10⁶/mm³). The highest RBC value (4.99 × 10⁶/mm³) was observed in the group receiving 100 mg/kg LAV, followed by other LAV-supplemented groups, indicating enhanced oxygen-carrying capacity. The hemoglobin levels also increased significantly (*P* = 0.001) in all LAV-treated groups compared to the control (10.19 g/dl). The 25 mg group recorded the highest Hb value (13.55 g/dl), reflecting enhanced blood oxygenation potential. There was a clear increase in PCV percentages across all treatment groups compared to the control (31.87%). The highest PCV was observed in birds receiving 150 mg/kg LAV (44.98%), indicating improved blood volume and overall physiological status.

**Table 6:** Impact of different levels of LAV supplementation on the hematological parameters of broiler chickens under Egyptian summer conditions

Parameters	Control	Aloe vera supplementation (mg)					SEM	*P*-value
		25	50	100	150	200		
RBCs (10^6^/mm^3^)	2.99^b^	4.78^a^	4.19^a^	4.99^a^	4.14^a^	3.89^a^	0.21	0.001
Hb (g/dL)	10.19^b^	13.55^a^	12.98^a^	13.09^a^	12.78^a^	13.01^a^	1.11	0.001
PCV (%)	31.87^b^	45.60^a^	40.90^a^	41.19^a^	44.98^a^	43.11^a^	3.03	0.0001

^a, b,^ means in the same row followed by different letters are significantly different at (*P* ≤ 0.05); SEM, Standard error of mean; LAV, lyophilized *Aloe vera*; RBCs, red blood cell; PCV, packed cell volume

### Blood biochemical parameters

#### Lipid profile

The data presented in Table 7 reveal that the inclusion of LAV in broiler diets exerted significant hypolipidemic effects. Total serum lipids were markedly reduced (*P* = 0.001) across all LAV-supplemented groups relative to the control (498.98 mg/dL), with the most pronounced decrease observed at the 200 mg/kg dosage (420.98 mg/dL). This dose-dependent reduction implies enhanced lipid catabolism or absorption modulation mediated by LAV. A similar trend was evident in cholesterol metabolism, where supplemented groups exhibited significantly lower concentrations (*P* = 0.002), declining from 199.12 mg/dL (control) to 177.89 mg/dL (100 mg/kg LAV).

**Table 7:** Impact of different levels of LAV supplementation on the lipid profile of broiler chicks under Egyptian summer conditions

Parameters	Control	Aloe vera supplementation (mg)					SEM	*P*-value
		25	50	100	150	200		
Total lipids (mg/dL)	498.98^a^	460.01^b^	455.09^b^	450.11^b^	400.91^b^	420.98^b^	15.10	0.001
Cholesterol (mg/dL)	199.12^a^	183.98^b^	180.11^b^	177.89^b^	181.12^b^	179.87^b^	6.09	0.002
Triglycerides (mg/dL)	141.51^a^	135.81^a^	134.92^a^	129.63^ab^	130.70^ab^	120.11^b^	2.77	0.001
HDL (mg/dL)	33.65^b^	38.98^a^	37.98^a^	39.98^a^	41.08^a^	40.11^a^	1.01	0.003
LDL (mg/dL)	137.17^a^	117.84^b^	115.15^b^	111.98^b^	113.90^b^	115.74^b^	1.29	0.001

^a, b,^ means in the same row followed by different letters are significantly different at (*P* ≤ 0.05); SEM, Standard error of mean; LAV, lyophilized *Aloe vera*; HDL, High density lipoprotein; LDL, Low density lipoprotein

Triglyceride levels followed this pattern (*P* = 0.001), diminishing from 141.51 mg/dL (control) to 120.11 mg/dL (200 mg/kg LAV). Notably, lipoprotein analysis revealed that low-density lipoprotein (LDL) levels decreased significantly (*P* = 0.001) in all supplemented groups, with the 150 mg/kg LAV group showing the most substantial reduction (113.90 mg/dL vs. 137.17 mg/dL in the control group). Conversely, HDL levels increased significantly (*P* = 0.003), peaking at 41.08 mg/dL and 40.11 mg/dL in the 150 mg/kg and 200 mg/kg LAV groups, respectively, compared to 33.65 mg/dL in the control group.

### Liver and renal functions

The control group exhibited significantly higher AST levels (67.11 U/L) compared to all LAV-supplemented groups (56.78–60.01 U/L; *P* = 0.001). The uniform reduction in AST across all LAV doses suggests that dietary Aloe vera supplementation enhances hepatic function by mitigating stress-induced enzyme leakage, likely attributable to its antioxidant or anti-inflammatory properties.

A similar trend was observed for ALT, with the control group showing elevated levels (42.56 U/L) relative to LAV-supplemented birds (37.98–39.01 U/L; *P* = 0.002). In contrast, ALP activity remained unchanged following LAV supplementation (*P* = 0.098), exhibiting only minor numerical variations across groups. These results indicate that *Aloe vera’s* protective effects are primarily targeted toward hepatocyte integrity rather than broader hepatic metabolic functions.

Table 8 demonstrates a significant reduction in serum creatinine levels (*P* = 0.002) across all LAV-treated groups compared to the control group (1.21 mg/dL). The most notable decreases were observed in the 50 mg and 200 mg LAV groups, both showing values of 0.876 mg/dL. These findings suggest improved kidney filtration, potentially linked to the antioxidant, and nephroprotective qualities of *Aloe vera*.

**Table 8:** Impact of different levels of LAV supplementation on liver and kidney functions of broiler chicks under Egyptian summer conditions

Parameters	Control	Aloe vera supplementation (mg)					SEM	*P*-value
		25	50	100	150	200		
AST (U/L)	67.11^a^	56.78^b^	55.98^b^	60.01^b^	58.01^b^	56.78^b^	1.23	0.001
ALT(U/L)	42.56^a^	38.90^b^	39.01^b^	38.04^b^	37.98^b^	39.00^b^	1.21	0.002
ALP(IU/L)	14.27	14.87	13.99	15.01	14.98	14.89	0.190	0.098
Creatinine (mg/dL)	1.210^a^	0.987^ab^	0.876^b^	0.901^b^	0.932^ab^	0.876^b^	0.078	0.002
Uric acid (mg/dL)	2.67^a^	1.56^b^	1.23^b^	1.43^b^	1.11^b^	1.09^b^	0.131	0.002

^a, b^ Means in the same row followed by different letters are significantly different at (*P* ≤ 0.05); SEM, Standard error of mean; LAV, lyophilized *Aloe vera*; ALT, alanine aminotransferase; AST, aspartate aminotransferase; ALP, Alkaline phosphatase

In a similar pattern, serum uric acid levels were substantially reduced (*P* = 0.002) in all treated groups relative to the control group (2.67 mg/dL). The observed range in supplemented groups was between 1.09 and 1.56 mg/dL, with the lowest levels detected in the 200 mg and 150 mg groups. These results point to enhanced nitrogen metabolism and renal function, likely resulting from the detoxifying properties of *Aloe vera*’s bioactive compounds.

### Glucose and thyroid hormones

The data presented in Table 9 demonstrate significant variations in plasma glucose concentrations and thyroid hormone profiles during the experimental period. Statistical analysis revealed markedly elevated (*P* = 0.001) blood glucose levels in LAVG-supplemented groups relative to the control group. At 35 days of age, dietary supplementation with LAV at concentrations of 25, 50, 100, 150, and 200 mg/kg feed resulted in respective increases of 17.2%, 18.5%, 15.7%, 11.9%, and 17.1% in plasma glucose levels compared to the control group.

**Table 9:** Impact of different levels of LAV supplementation on blood glucose and thyroid hormones of broiler chicks under Egyptian summer conditions

Parameters	Control	Aloe vera supplementation (mg)					SEM	*P*-value
		25	50	100	150	200		
Glucose (mg/dL)	151.98^b^	178.09^a^	180.03^a^	175.87^a^	170.06^a^	177.98^a^	4.15	0.001
T3(ng/dL)	2.01^c^	3.76^a^	3.89^a^	2.99^b^	3.38^a^	3.20^a^	0.12	0.002
T4(ng/dL)	6.89^b^	9.98^a^	10.08^a^	9.01^a^	9.67^a^	10.31^a^	0.076	0.0001
T3/T4	0.291	0.376	0.286	0.331	0.349	0.310	0.043	0.098

^a, b,c^ means in the same row, followed by different letters are significantly different at (*P* ≤ 0.05); SEM, Standard error of mean; LAV, lyophilized *Aloe vera*; T3, Triiodothyronine; T4, Thyroxin

The experimental results revealed significant elevations in thyroid hormone concentrations following LAV supplementation. Plasma T3 levels increased marked (*P* = 0.002) across all LAV-treated groups compared to the control. At 35 days of age, T3 concentrations were elevated by 87.1%, 93.5%, 48.8%, 98.0%, and 94.0% in the respective LAV treatment groups. Similarly, plasma thyroxine (T4) levels demonstrated significant enhancement (*P* = 0.0001), with corresponding increases of 73.9%, 59.4%, 74.3%, 83.9%, and 88.8% observed in the LAV-supplemented groups. However, statistical analysis indicated no significant differences between treatments (*P* > 0.05) on the T3/T4 ratio throughout the experimental period.

### Antioxidant indices

Table 10 illustrates the effect of various dietary levels of LAV supplementation on key antioxidant markers. TAC concentration, which measures the overall ability of plasma to combat oxidative stress, was also significantly elevated in all treated groups (400.5–425.9 nmol/L) compared to the control (377.9 nmol/L). The highest TAC level was recorded in the 200 mg LAV group, demonstrating Aloe vera’s effectiveness in enhancing systemic antioxidant capacity. The activity of SOD, a crucial antioxidant enzyme responsible for shielding cells from oxidative damage, showed a significant increase (*P* = 0.001) across all LAV-supplemented groups compared to the control (191.89 U/mL). The highest SOD levels were observed in the 150 mg (254.8 U/mL) and 200 mg (245.9 U/mL) LAV groups, indicating an improved enzymatic defense mechanism against reactive oxygen species (ROS).

**Table 10:** Impact of different levels of LAV supplementation on indicators of antioxidative status in the blood of broiler chicks under Egyptian summer conditions

Parameters	Control	Aloe vera supplementation (mg)					SEM	*P*-value
		25	50	100	150	200		
TAC (nmol/L)	377.9^b^	420.7 ^a^	400.5 ^a^	412.8 ^a^	420.0 ^a^	425.9 ^a^	11.99	0.001
SOD (U/ml)	191.89^b^	210.7^ab^	220.9^ab^	239.8 ^a^	254.8 ^a^	245.9 ^a^	4.65	0.001
GSH-Px(mmol/L)	790.1^b^	820.7 ^a^	830.3 ^a^	845.8 ^a^	855.3 ^a^	840.6 ^a^	22.12	0.001
GSH (mmol/L)	0.400^b^	0.512 ^a^	0.480 ^a^	0.530 ^a^	0.550 ^a^	0.560 ^a^	0.04	0.001
MDA (nmol/L)	166.9 ^a^	144.8^b^	140.9^bc^	139.1^bc^	122.9^c^	135.0^bc^	3.53	0.003

^a, b,c^ means in the same column followed by different letters are significantly different at (*P* ≤ 0.05); SEM, Standard error of mean; LAV, lyophilized *Aloe vera*; TAC, total antioxidant capacity; SOD, superoxide dismutase; GSH-Px, glutathione peroxidase; GSH, glutathione; MDA, malondialdehyde

The activity of GSH-Px, a key enzyme that neutralizes harmful peroxides, was significantly increased (*P* = 0.001) in all LAV-treated groups compared to the control group (790.1 mmol/L). The highest enzymatic activity was observed in the group receiving 150 mg LAV, reaching 855.3 mmol/L, reflecting a strengthened enzymatic antioxidant defense under heat stress conditions. Similarly, levels of reduced GSH, an essential non-enzymatic antioxidant, showed a significant elevated in all supplemented groups compared to the control (0.400 mmol/L). The most notable increase was occurred in the 200 mg group (0.560 mmol/L), indicating improved intracellular redox capacity and enhanced cellular antioxidant reserves.

In contrast, MDA, a marker of lipid peroxidation and oxidative damage, significantly decreased (*P* = 0.003) in all treated groups. The highest reduction was observed at 150 mg (122.9 nmol/L), compared to the control value of 166.9 nmol/L, suggesting reduced oxidative damage to cell membranes.

## Discussion

### Growth performance

Heat stress during the Egyptian summer represents a major obstacle in broiler production due to prolonged exposure to high ambient temperatures and relative humidity, leading to compromised growth performance, metabolic disturbances, and impaired physiological homeostasis. In this study, the recorded THI equal 29.54 exceeded the critical threshold for poultry, confirming exposure to moderate to severe heat stress and validating the experimental conditions ( Hemida et al. 2023). Similar thermal stress scenarios under tropical conditions have recently been reported to hurt productivity and oxidative balance in broiler chickens (Abdel-Fattah et al. 2024; Abd El-Ghany, 2024). Under these adverse conditions, dietary supplementation with LAV improved productive performance and physiological responses, supporting its role as a functional phytogenic additive. Recent evidence suggests that phytogenic feed additives rich in bioactive compounds can improve thermotolerance, antioxidant capacity, and immune resilience in heat-stressed broilers (Abd El-Ghany, 2024a, b). The beneficial effects of Aloe vera are largely attributed to its complex phytochemical profile, which includes polysaccharides (acemannan), flavonoids, phenolic acids, vitamins, and trace minerals, which together regulate digestion, immunity, and redox balance (Hamman 2008; Sharma et al. 2014).

Nevertheless, the absence of proportional improvements at higher inclusion levels observed in this study suggests a dose-dependent response. This is consistent with recent findings indicating that excessive phytogenic supplementation may disrupt metabolic balance rather than improve performance, particularly under environmental stress (Gadde et al. 2017; Abd El-Ghany 2024c). Wenk (2003) and Samarasinghe et al. (2003) reported that the inclusion of herbs in poultry diets can stimulate appetite and enhance endogenous secretions, thereby supporting improving physiological function. The antibacterial and antioxidant properties of AV, as demonstrated by Arunkumar and Muthuselvam (2009), may help explain the observed improvements in growth performance and the favorable FCR in AV-treated broilers.

These findings are consistent with the report by Changkang et al. (2007), who found that supplementation with AV gel water extract significantly increased BW in broilers. Similarly, Mehala and Moorthy (2008) observed that the inclusion of 1% AV leaf meal in broiler diets led to a significant increase in BW and overall growth compared to un-supplemented control group. Durrani et al. (2008) attributed enhanced BWG and improved FCR in AV-treated birds to superior performance and the diverse antimicrobial activities of AV gel. Furthermore, Fallah (2015) reported that the inclusion of 1.5% AV in drinking water led to increased feed consumption. This observation is supported by Shokraneh et al. (2016), who noted a significant rise in daily feed intake following the addition of 1% AV gel to drinking water, compared to the control group during the experimental period.

Also, Islam et al. (2019) reported that broilers provided with drinking water containing 15 mL/L of AV gel exhibited increased feed intake, which was attributed to the phytogenic compounds present in AV that stimulate appetite and enhance overall performance. Similarly, Ebrahim et al. (2020) associated the rise in feed consumption among AV-supplemented birds with improved palatability, appetite stimulation, enhanced secretion of pancreatic and digestive enzymes, better nutrient digestibility, and increased intestinal absorptive capacity.

Consistent with the current study’s results, several previous investigations have reported beneficial effects of AV supplementation on performance parameters in poultry. Odo et al. (2010) observed zero mortality in cockerels at 72 days of age across all AV-treated groups. The beneficial effects of AV gel, which is rich in bioactive compounds such as vitamins, minerals, enzymes, organic acids, and carbohydrates, may contribute to enhanced broiler performance. Yim et al. (2011) reported that birds receiving AV supplementation exhibited improved FCR compared to control group. Surjushe et al. (2008) noted that AV is a rich source of biologically active compounds, including proteins, carbohydrates, vitamins, enzymes, minerals, sugars, lignins, saponins, and salicylic acid, which may collectively support growth and health. Olupona et al. (2010) demonstrated that administering AV gel mixed with drinking water significantly increased both final and weekly BW in broilers. According to Darabighane et al. (2012), AV gel extracts not only provide antimicrobial agents but also essential nutrients, which likely contributed to increased final BW, weekly weight gain, and improved FCR.

Although phytogenic feed additives generally show beneficial effects, their efficacy may vary due to differences in the concentration and composition of active phytochemicals (Gadde et al. 2017). The mechanisms of action of these bioactive compounds remain complex and are not yet fully understood. Notably, the current results suggest that excessive inclusion of Aloe vera gel may negatively affect broiler productivity. This underscores the limited research on the potential toxic effects of Aloe vera, which may depend on factors like season, location, irrigation, harvest time, and the lack of standardized gel processing methods (Xiang et al. 2017). Aloe vera, known for its richness in free anthraquinones and their derivatives, has been reported to enhance intestinal absorption and exert strong antibacterial activity. Additionally, supplementing with LAV has been shown to promote cellular proliferation (Talmadge et al. 2004). Similarly, Womble and Helderman (1988) observed that AV encouraged the growth of new cells, leading to increased body weight in treated animals. According to Islam et al. (2019), the flavonoid content of Aloe barbadensis helps improve broiler live body weight by modulating intestinal microflora, boosting metabolic efficiency, and increasing glycogen storage in liver and muscle tissues.

Furthermore, Aloe vera is widely recognized as a medicinal plant with a broad range of biological activities, including anti-inflammatory (Langmead et al. 2004), antioxidant (El-Shemy et al. 2010), antimicrobial, antiseptic, analgesic, and immunomodulatory effects (Sharma et al. 2014), all of which may contribute to better physiological functions and overall health in poultry. The enhancements in growth performance observed in the present study as a result of dietary supplementation with AV powder are consistent with the findings of Mohamed and Hassan (2022), who demonstrated the efficacy of phytogenic compounds as safe and effective growth promoters in poultry nutrition. Similarly, Arif et al. (2022) reported that incorporating of AV leaf powder at levels of 0.5% and 1.0% in the diets of Fayoumi chicks and quails significantly improved final BW and BWG compared to the control group. In addition, Kichloo et al. (2023) indicated that AV gel extract is rich in bioactive compounds, including essential nutrients and antimicrobial constituents, which may collectively contribute to improved FC, higher weekly growth rates, and enhanced overall weight gain in poultry.

### Apparent digestibility

Improvements in growth performance and feed conversion ratio appear to be primarily mediated through increased digestibility and nutrient utilization rather than simply increases in feed intake. The bioactive polysaccharides and saponins present in Aloe vera can improve the intestinal absorption capacity by stabilizing the intestinal microbiota, preserving epithelial integrity, and stimulating secretion of endogenous digestive enzymes, thereby improving protein and lipid digestibility (Abdel-Moneim et al. 2020; Abd El-Ghany 2025b). This mechanism is particularly relevant during heat stress, where gastrointestinal function is typically impaired.

The increase in digestibility of crude protein, ether extract, and dry matter seen here supports better gastrointestinal functionality. During heat stress, lipid digestion is often affected due to reduced bile secretion and enzyme activity (Quinterio-Filho et al. 2010). Therefore, improved ether extract digestibility after LAV supplementation may reflect stimulated bile flow and lipase activity, leading to more efficient lipid utilization (Abd El-Ghany 2025a). The lack of significant changes in crude fiber and ash digestibility suggests limited response of these fractions to phytogenic modulation, as previously reported (Alagawany et al. 2021).

### Hematological parameters

Hematological indices are sensitive indicators of physical and health status. The observed increases in red blood cell count, hemoglobin concentration, and packed cell volume at moderate LAV inclusion levels indicate improved erythropoiesis and oxygen-carrying capacity, which are important for maintaining metabolic activity under thermal stress (Soetan et al. 2013). These effects are likely linked to aloe vera’s vitamin and mineral-rich composition, which includes iron, folic acid, and B-complex vitamins, which support hematopoietic function (Kayode 2017). In conclusion, dietary LAV supplementation, particularly at 100–150 mg/kg, significantly improved RBC count, hemoglobin concentration, and PCV, which are key indicators of enhanced physiological and health status in broilers, supporting the role of LAV as a functional feed additive under high temperature conditions.

Hamman (2008) reported that AV contains essential vitamins and amino acids required for Hb synthesis, which contribute to improvements in hematological parameters. Similarly, Mmereole (2011) found that birds supplemented with AV exhibited superior blood profiles compared to those receiving conventional antibiotic treatments. The hematological benefits observed in this study may be attributed, in part, to the diverse and bioactive nutrient profile of *Aloe vera*. Kayode (2017) attributed the beneficial effects of AV to its rich composition of vitamins-including β-carotene, vitamins C, E, B12, riboflavin, thiamine, and folic acid-as well as essential trace minerals such as calcium, chromium, copper, selenium, manganese, potassium, sodium, and zinc. These micronutrients are known to support erythropoiesis and overall hematological health. Moreover, Yadav et al. (2017) observed that chicks receiving AV juice in their drinking water displayed elevated hematological indices, including higher mean corpuscular hemoglobin concentration, suggesting that AV extract may be a potential therapeutic agent for iron-deficiency anemia.

### Blood biochemical parameters

#### Lipid profile

These findings suggest that dietary supplementation with LAV at levels of 150–200 mg/kg effectively modulates lipid metabolism, as evidenced by a reduction in total cholesterol, triglycerides, and LDL cholesterol, along with an increase in HDL cholesterol. This modulation may contribute to improved metabolic health in broiler chickens. Dietary supplementation of LAV also regulated lipid metabolism, as evidenced by elevated HDL levels as well as reduced serum total cholesterol, triglyceride, and LDL concentrations. These findings are consistent with recent reports showing hypolipidemic effects of phytogenic additives through improved insulin sensitivity and hepatic lipid regulation (Abd El-Ghany 2024b,c). Acemannan, a major polysaccharide in Aloe vera gel, has been shown to regulate hepatic lipid synthesis and lipoprotein metabolism, which may explain the observed lipid profile improvements (Misawa et al. 2012). The current results are consistent with those of Amber et al. (2021), who reported a significant reduction in blood cholesterol, triglycerides, and LDL levels, accompanied by elevated HDL concentrations in birds receiving 150–200 mg of Aloe vera gel in their diets compared to the control group. Taraneh (2016) observed that broilers provided with 3% *Aloe vera* solution in drinking water exhibited significant decreases in uric acid, total cholesterol, LDL, and triglyceride concentrations.

### Liver and renal functions

The reduction in serum AST and ALT activities further indicates the hepatoprotective effects of LAV supplementation. Heat stress is known to induce oxidative damage to hepatocyte membranes, leading to increased leakage of enzymes into the circulation. The antioxidant and anti-inflammatory properties of Aloe vera probably help preserve hepatocellular integrity and limit liver stress (Boniface et al. 2023; Abd El-Ghany 2024a). Furthermore, renal function markers remained within physiological limits, suggesting that LAV supplementation did not impose a metabolic burden on the renal clearance mechanism, which is consistent with recent safety evaluations (Arif et al. 2022; El-Kholy et al. 2022).

Urea and creatinine are nitrogenous waste products eliminated from the body primarily through renal excretion. Efficient elimination of these metabolites not only reflects optimal kidney function but also suggests enhanced metabolic efficiency, which may contribute to improved body weight and overall productivity. In the current study, dietary supplementation with Aloe vera (AV), particularly at doses ranging from 50 to 200 mg/kg, effectively maintained renal function markers within or near physiological ranges, even under heat stress conditions. These findings suggest that Aloe vera supplementation supports renal health and systemic metabolic stability in broilers exposed to thermal stress.

### Glucose and thyroid hormones

The observed enhancements in blood glucose and thyroid hormone levels in broilers supplemented with LAV likely contributed to the improved BWG, despite variations in feed intake. Thyroid hormones are central to metabolic regulation, notably increasing basal metabolic rate and influencing protein, carbohydrate, and lipid metabolism. Their role in mobilizing and utilizing lipid reserves, particularly by stimulating triglyceride breakdown in adipose tissue, may explain the reductions in serum lipid profiles seen in LAV-treated birds. Moreover, blood glucose levels may serve as indicators of metabolic activity and physiological adaptation, which are critical for growth and stress resilience in commercial broiler production systems (Melesse et al. 2011). Supporting these findings, Singh et al. (2013) reported that broilers provided with 20 g/L of fresh Aloe vera juice via drinking water exhibited significantly elevated plasma glucose concentrations compared to control birds. Together, these results suggest that LAV supplementation may enhance endocrine and metabolic efficiency, contributing to improved performance under thermally stressful conditions.

### Antioxidant indices

Oxidative stress mitigation represents a central mechanism underlying the observed benefits of LAV under heat stress. Enhanced activities of superoxide dismutase, glutathione peroxidase, total antioxidant capacity, and reduced glutathione, coupled with decreased malondialdehyde concentrations, indicate improved redox homeostasis. These effects are attributed to phenolics, flavonoids, and chromone derivatives in Aloe vera with strong free radical–scavenging activity (Sharma et al. 2018; Abd El-Ghany 2024a). Oxidative stress arises from the accumulation of ROS and harmful peroxides, such as hydrocarbons, aldehydes, and ketones, which can degrade vitamins and carotenoids and potentially exert mutagenic or carcinogenic effects (Mazur-Kuśnirek et al. 2019). The body’s antioxidant defense system, consisting of enzymatic components like SOD and GSH-Px, as well as non-enzymatic elements such as reduced GSH. *Aloe vera* rich composition, including vitamins, enzymes, minerals, polysaccharides, and phenolics, underpins its health benefits (Tabrizi and Mohajeri 2012). The high phenolic content found in AV leaf skin correlates positively with antioxidant capacity (López et al. 2013), with its polyphenolic compounds have been shown to combat oxidative stress and inhibit lipid peroxidation (Sharma et al. 2018). Consistently, Amber et al. (2021) found that broilers supplemented with 1.5% AV gel had enhanced total antioxidant capacity and GSH-Px activity, along with reduced MDA levels.

Recent studies support the immunomodulatory role of medicinal plants in broilers. Hussain et al. (2023) demonstrated that *Artemisia brevifolia* extract significantly improved cellular and humoral immune responses and reduced intestinal lesion scores in coccidia-challenged broilers. Similarly, Abbas and Alkharaije (2023) reported that *Carica papaya* extract improved immune regulation and intestinal elasticity against experimental coccidiosis. Furthermore, Al-Hoshani et al. (2023) found that *Illicium verum* essential oil exerts anticoccidial and immunoprotective effects through antioxidant and anti-inflammatory mechanisms. These findings support the interpretation that the physiological benefits of LAV supplementation observed in this study may be partially mediated by immune stabilization and reduction of inflammatory burden.

Overall, the present findings demonstrate that lyophilized aloe vera supplementation improves growth performance, physiological stability, immunogenicity, and antioxidant capacity in heat-stressed broiler chickens through integrated digestive, immunological, metabolic, and antioxidant mechanisms. These results support the use of Aloe vera as a natural feeding strategy to reduce heat stress in poultry production under tropical climate conditions.

## Conclusion

In this study, dietary supplementation with freeze-dried aloe vera was associated with dose-dependent improvements in growth performance and selected physiological indicators in broilers exposed to heat stress. The findings suggest possible beneficial effects on improved antioxidant status as well as liver and kidney function, which may contribute to better adaptation to higher ambient temperatures. Supplementation levels of 150 and 200 mg/kg showed more consistent responses under Egyptian summer environmental conditions. However, these results must be interpreted within the limitations of the experimental design. Further studies are needed to clarify the underlying mechanisms and assess long-term effects on health, productivity and other quality characteristics.

## Data Availability

N/A.
